# Complete chloroplast genome comparisons for *Pityopsis* (Asteraceae)

**DOI:** 10.1371/journal.pone.0241391

**Published:** 2020-12-28

**Authors:** E. Anne Hatmaker, Phillip A. Wadl, Timothy A. Rinehart, Jennifer Carroll, Thomas S. Lane, Robert N. Trigiano, Margaret E. Staton, Edward E. Schilling

**Affiliations:** 1 Department of Entomology and Plant Pathology, University of Tennessee, Knoxville, Tennessee, United States of America; 2 U.S. Department of Agriculture, Agricultural Research Service, U.S. Vegetable Laboratory, Charleston, South Carolina, United States of America; 3 U.S. Department of Agriculture, Agricultural Research Service, Crop Production and Protection, Beltsville, Maryland, United States of America; 4 U.S. Department of Agriculture, Agricultural Research Service, Thad Cochran Southern Horticultural Laboratory, Poplarville, Mississippi, United States of America; 5 Department of Ecology and Evolutionary Biology, University of Tennessee, Knoxville, Tennessee, United States of America; National Cheng Kung University, TAIWAN

## Abstract

*Pityopsis* includes several regionally and one federally endangered species of herbaceous perennials. Four species are highly localized, including the federally endangered *P*. *ruthii*. The genus includes several ploidy levels and interesting ecological traits such as drought tolerance and fire-dependent flowering. Results from previous cladistic analyses of morphology and from initial DNA sequence studies did not agree with one another or with the infrageneric taxonomic classification, with the result that infrageneric relationships remain unresolved. We sequenced, assembled, and compared the chloroplast (cp) genomes of 12 species or varieties of *Pityopsis* to better understand generic evolution. A reference cp genome 152,569 bp in length was assembled *de novo* from *P*. *falcata*. Reads from other sampled species were then aligned to the *P*. *falcata* reference and individual chloroplast genomes were assembled for each, with manual gapfilling and polishing. After removing the duplicated second inverted region, a multiple sequence alignment of the cp genomes was used to construct a maximum likelihood (ML) phylogeny for the twelve cp genomes. Additionally, we constructed a ML phylogeny from the nuclear ribosomal repeat region after mapping reads to the *Helianthus annuus* region. The chloroplast phylogeny supported two clades. Previously proposed clades and taxonomic sections within the genus were largely unsupported by both nuclear and chloroplast phylogenies. Our results provide tools for exploring hybridity and examining the physiological and genetic basis for drought tolerance and fire-dependent flowering. This study will inform breeding and conservation practices, and general knowledge of evolutionary history, hybridization, and speciation within *Pityopsis*.

## Introduction

*Pityopsis* is a small genus of Asteraceae with its center of diversity in the southeastern United States [1]. The genus includes a wide variety of ploidy levels across species and a large geographic range throughout southeastern North America, in Mexico and Central America, and in the Bahamas [2]. Notably, four species of *Pityopsis* are rare and of conservation concern: *P*. *ruthii* (listed as endangered federally), *P*. *flexuosa* (listed as endangered by the state of Florida), *P*. *falcata* (listed as endangered by the state of Connecticut and of special concern by the state of Rhode Island), and *P*. *pinifolia* (listed as threatened by the state of Georgia). *Pityopsis* has been the subject of several phylogenetic studies [3–5], but intrageneric relationships for all species and varieties in the genus have not been fully resolved, resulting in significant variation in the number of species recognized within *Pityopsis*. The genus includes many polyploid varieties and several ecologically adaptive traits such as fire-stimulated flowering [3,6] and drought-tolerance [7]. Studying species relationships often allows for better evolutionary understanding of traits.

Phylogenetic studies are conducted to clarify taxonomic relationships and classification [8]. They have proved useful for understanding plant-pathogen interactions [9] and community ecology [10]. Additionally, phylogenetic studies can translate to predictions of phenological response and adaptation in related species, especially adaptation in regard to climate change [11]. Phylogenies have additional use in studies focused on evolutionary history [12]. *Pityopsis* is an excellent candidate for such analysis as the genus includes species that vary for traits such as fire-adaptive flowering, as well as species with varying ploidy levels [4]. In *Pityopsis*, species distinctions are not well understood and require further resolution, which has been difficult due to the differing ploidy levels in the genus and apparent hybridization. For example, in *P*. *graminifolia* alone there are three ploidy levels present in different varieties of the species: diploid (*P*. *graminifolia* var. *graminifolia*), tetraploid (var. *latifolia*), and hexaploid (var. *tracyi*) [13]. Analyzing datasets with a range of ploidy levels creates difficulties when using biparental nuclear markers. However, with a well-supported phylogeny based on molecular markers, *Pityopsis* could be used to examine the evolution of adaptive traits and the role of hybridity in the evolution of polyploidy.

Nuclear microsatellites have been developed for two different *Pityopsis* species and chloroplast microsatellites have been developed for one species [14–16]. However, whole chloroplast (cp) genomes are lacking for all species in the genus. With the availability of next-generation sequencing, phylogenetic studies using entire cp genomes is becoming more reliable and common, especially for closely related species [17]. Chloroplast genome sequences have become a convenient way to find repetitive sequences and single nucleotide polymorphisms (SNPs) that could be used for further ecological and evolutionary studies, as well as clarifying taxonomy in genera with muddled history [18]. Many similar studies have been conducted on phylogenetic relationships within economically important plants, such as wheat, rice, and maize [19], strawberry [20], and cotton [21]. Using cp genomes to analyze the species relationships within *Pityopsis* allows further studies regarding past polyploid events to use a simplified system due to the haploid nature of chloroplasts, though only the maternal line is revealed in the case of species arising from hybridization events resulting in allopolyploidy.

*Pityopsis* includes seven species: *P*. *aspera* (Shuttlew. ex Small) Small, *P*. *falcata* (Pursh) Small, *P*. *flexuosa* (Nash) Small, *P*. *graminifolia* (Michx.) Nutt., *P*. *oligantha* (Chapm. ex Torr. & Gray) Small, *P*. *pinifolia* (Ell.) Nutt., and *P*. *ruthii* (Small) Small [15]. Both *P*. *aspera* and *P*. *graminifolia* have multiple varieties, some of which have previously been recognized as separate species [22]. *Pityopsis* is endemic to the eastern United States, and though *P*. *graminifolia* and *P*. *aspera* have a large range, other species in the genus are more localized, such as *P*. *ruthii* and *P*. *flexuosa*. All species are perennial and have yellow inflorescences, as indicated by the common name for plants in the genus, goldenaster [13].

The division of *Pityopsis* into sections remains unresolved. Semple and Bowers [13], divided the genus into two sections: section *Pityopsis* with *P*. *falcata*, *P*. *flexuosa*, *P*. *pinifolia*, and *P*. *ruthii*, and section *Graminifoliae* with *P*. *aspera*, *P*. *graminifolia*, and *P*. *oligantha*. However, the phylogenetic analysis conducted by Gowe and Brewer [3] based on morphology divided the species into two clades that did not coincide with the sectional classification, and were referred to informally as the Falcata clade, which includes *P*. *falcata*, *P*. *flexuosa*, *P*. *graminifolia*, *P*. *pinifolia*, and *P*. *oligantha*, and the Aspera clade, which includes *P*. *aspera*, *P*. *adenolepis*, and *P*. *oligantha*. In contrast, a molecular study utilized sequences from chloroplast and nuclear regions of all seven species and concluded that two new clades should be named: Ruthii and Flexuosa [4]. Clade Ruthii includes *P*. *falcata*, *P*. *pinifolia*, *P*. *ruthii*, and *P*. *graminifolia* var. *latifolia*. Splitting the species *P*. *graminifolia*, clade Flexuosa includes *P*. *graminifolia* var. *aequilifolia*, *P*. *graminifolia* var. *tenuifolia*, and *P*. *graminifolia* var. *graminifolia*, as well as *P*. *aspera*, *P*. *adenolepis*, and *P*. *oligantha*. Both the 2005 and the 2008 studies include *P*. *adenolepis* as a separate species from *P*. *aspera* as per Clewell [22], although Nesom [2] considers them synonymous based on his interpretation of morphology. We have continued to use the taxonomic designations set forth by Semple and Bowers [13] as there is no agreement on naming of varieties or species even as recently as 2019 [2,23]. With little to no consensus between morphological and molecular studies, any information derived from molecular studies within the genus can only improve taxonomic resolution.

In this study, 12 *Pityopsis* chloroplast genomes were assembled, compared to other Asteraceae chloroplast genomes, and used to construct phylogenetic trees. To provide additional information from the biparentally inherited nuclear genome, we also constructed phylogenetic trees using the nuclear external transcribed spacer (ETS) region, which is highly conserved, to complement the chloroplast phylogenies and to add to our knowledge of hybridity and evolution of the *Pityopsis* genus. Although resolving the taxonomic problems of the genus is beyond the scope of the current study, here we present data on chloroplast genomes that will provide a foundation for future studies of *Pityopsis*.

## Methods

### Ethics statement for plant collection

Leaf tissue of seven species including seven varieties of *Pityopsis* was collected from the southeastern United States (Table 1). Leaf tissue from plants maintained in a greenhouse at the University of Tennessee was collected for *P*. *graminifolia* var. *tracyi*. This study used tissue collected in 2010 and 2013 and kept at -80°C from *P*. *ruthii* [14], *P*. *falcata*, and *P*. *graminifolia* var. *latifolia* [15], respectively. Original plant material for *P*. *ruthii* and *P*. *graminifolia* var. *latifolia* was collected in Polk County, Tennessee under permits from the Tennessee Valley Authority (TE117405-2) and the United States Fish and Wildlife Service (TE134817-1). All other tissue was collected from public land which required no permit, under permit from the South Carolina Department of Natural Resources, or in coordination with United States Forest Service scientists. *Pityopsis aspera* var. *adenolepis*, *P*. *graminifolia* var. *tenuifolia*, and *P*. *graminifolia* var. *graminifolia* were collected from Florence and Darlington counties in South Carolina in 2014. Tissue for *P*. *graminifolia* var. *aequilifolia* was collected in Ocala National Forest in 2015. For *P*. *oligantha* and *P*. *flexuosa*, tissue was collected in 2014 and 2015 from Liberty and Wakulla counties, respectively, in Florida. Tissue was collected in 2016 for *P*. *aspera* var. *aspera* in Florida and *P*. *pinifolia* in the Peachtree Rock Heritage Preserve in Lexington County, South Carolina. Vouchers are available at the Florida State University Herbarium (FSU) for *P*. *oligantha* and *P*. *flexuosa* (Anderson 28905 and Anderson 28533, respectively).

**Table 1:** State of tissue after collection, date, and location of *Pityopsis* individuals.

Species	Type of tissue	Year collected	Location
*P*. *aspera* var. *adenolepis*	Dried	2014	South Carolina
*P*. *aspera* var. *aspera*	Dried	2016	Florida
*P*. *falcata*	Dried	2010	Rhode Island
*P*. *flexuosa*	Dried	2015	Florida
*P*. *graminifolia* var. *aequilifolia*	Dried	2015	Florida
*P*. *graminifolia* var. *graminifolia*	Frozen	2014	South Carolina
*P*. *graminifolia* var. *latifolia*	Frozen	2013	Tennessee
*P*. *graminifolia* var. *tenuifolia*	Frozen	2014	South Carolina
*P*. *graminifolia* var. *tracyi*	Fresh	2014	Florida
*P*. *oligantha*	Dried	2015	Florida
*P*. *pinifolia*	Dried	2016	South Carolina
*P*. *ruthii*	Frozen	2010	Tennessee

### Library construction and sequencing

Total genomic DNA (gDNA) was isolated using a DNeasy Plant Mini Kit (Qiagen, Valencia, CA) following manufacturer’s protocol. Genomic DNA of all samples was cleaned and concentrated using the Zymo Genomic DNA Clean and Concentrator Kit (Zymo Research Corp., Irvine, CA). The libraries were prepared using the Nextera DNA Library Preparation Kit (Illumina, San Diego, CA). DNA was fragmented using transposase-mediated tagmentation and paired end sequenced using dual indexes. The Illumina MiSeq version 3 sequencing platform (Illumina, San Diego, CA) was used for 250 bp paired-end sequencing of the DNA. Three libraries were pooled for three runs and four pooled for one run. One run was discarded due to low-quality.

### Sequence trimming and alignment

The sequence quality of all sequences was checked using FastQC [24] for kmer content, GC content, and average length of reads. Adaptors and low quality ends were trimmed using Trimmomatic v. 0.35 [25]. After trimming, quality was assessed again using FastQC, which showed that overall quality improved in all individuals. Using the program Bowtie2 [26], the data from all individuals was aligned against the chloroplast genome of *Helianthus annuus*, which was downloaded from NCBI (GenBank: DQ383815.1; downloaded November, 2015). *P*. *falcata* had the highest number of mapped reads after the first round of sequencing, and was therefore selected for *de novo* assembly of a reference cp genome.

### Genome assembly and annotation

After mapping *P*. *falcata* reads to the *H*. *annuus* chloroplast genome to filter out genomic DNA, the *P*. *falcata* reads were assembled into a reference cp genome using the program ABySS v 1.5.2 [27], which is designed for short, paired-end reads. Gaps within the draft genome were closed using the “map to reference” option within Geneious 11.1.5 [28] using *P*. *falcata* reads and default parameters. Additionally, *P*. *falcata* reads were mapped back to the *P*. *falcata* reference cp genome to fill short gaps and call variants. Reads from each individual species and variety were mapped to the *P*. *falcata* reference to generate a consensus sequence, which served as a draft cp genome. The draft genomes were then gapfilled within Geneious [28] using the “map to reference” option. We also assembled a cp genome from a second *P*. *ruthii* individual for quality control using sequencing data from previous work [14] (S1–S3 Tables) and the same methodology.

The reference genome from *P*. *falcata* was annotated using DOGMA [29], which is specific to organelle genomes and also identifies tRNAs and rRNAs. The annotations were manually reviewed and edited within Geneious v. 11.1.5 [28]. Visualization of the genome annotation as a gene map was created using the program OGDraw [30].

### Alignment and comparison

The *Pityopsis* and *H*. *annuus* cp genomes (after removal of the duplicate copy of the inverted region) were then aligned using Mauve [31]. Pairwise differences were calculated between all *Pityopsis* cp genomes and the outgroup *H*. *annuus* cp genome within Mauve [32]. The substitution model was chosen using the corrected Akaike information criterion (AICc) as calculated by JModelTest [33]. The maximum likelihood cp phylogenetic tree was built using RAxML 8.2.11 [34] GTR + GAMMA + I parameter within RAxML [34]. Bootstrap analysis was conducted using 1000 replicates. The consensus tree was drawn with a 10% burn-in and 50% support threshold.

Using Geneious [28], gDNA reads of all 12 *Pityopsis* individuals were mapped to the 9814 bp nuclear ribosomal repeat region of *H*. *annuus* (KF767534.1) including the 28S, 18S, and 5.8S genes. Consensus sequences were called and gDNA mapped back to the consensus sequences for all *Pityopsis* taxa. The nuclear ribosomal region of *H*. *annuus* was used as an outgroup and aligned along with the *Pityopsis* ribosomal regions using MUSCLE [35] with anchor optimization, and the substitution model selected using AICc as previously outlined. A maximum likelihood phylogeny was reconstructed in RAxML [34] using the GTR + GAMMA parameter with 1000 replicates for bootstrapping. The consensus tree was drawn with a 10% burn-in and 50% support threshold.

## Results

### Chloroplast genome sequencing, assembly, and annotation

Using the Illumina MiSeq sequencing platform, we sequenced gDNA and assembled cp genomes for 12 samples representing 7 species from *Pityopsis*, including 7 varieties (TtT 1). Illumina paired-end sequencing produced from 3,451,455 (*P*. *oligantha*) to 33,339,900 (*P*. *graminifolia* var. *aequilifolia*) reads per individual (S1 Table). Of these reads, 6,571 (*P*. *graminifolia* var. *aequilifolia*) to 199,621 (*P*. *ruthii*) reads mapped to the *H*. *annuus* reference cp genome, with 5–240 x coverage (S2 Table). *P*. *aspera* var. *aspera* had the highest number of basepairs mapped to the *Helianthus* reference (S2 Table), whereas *P*. *graminifolia* var. *aequilifolia* had the fewest mapped reads.

**Fig 1:**
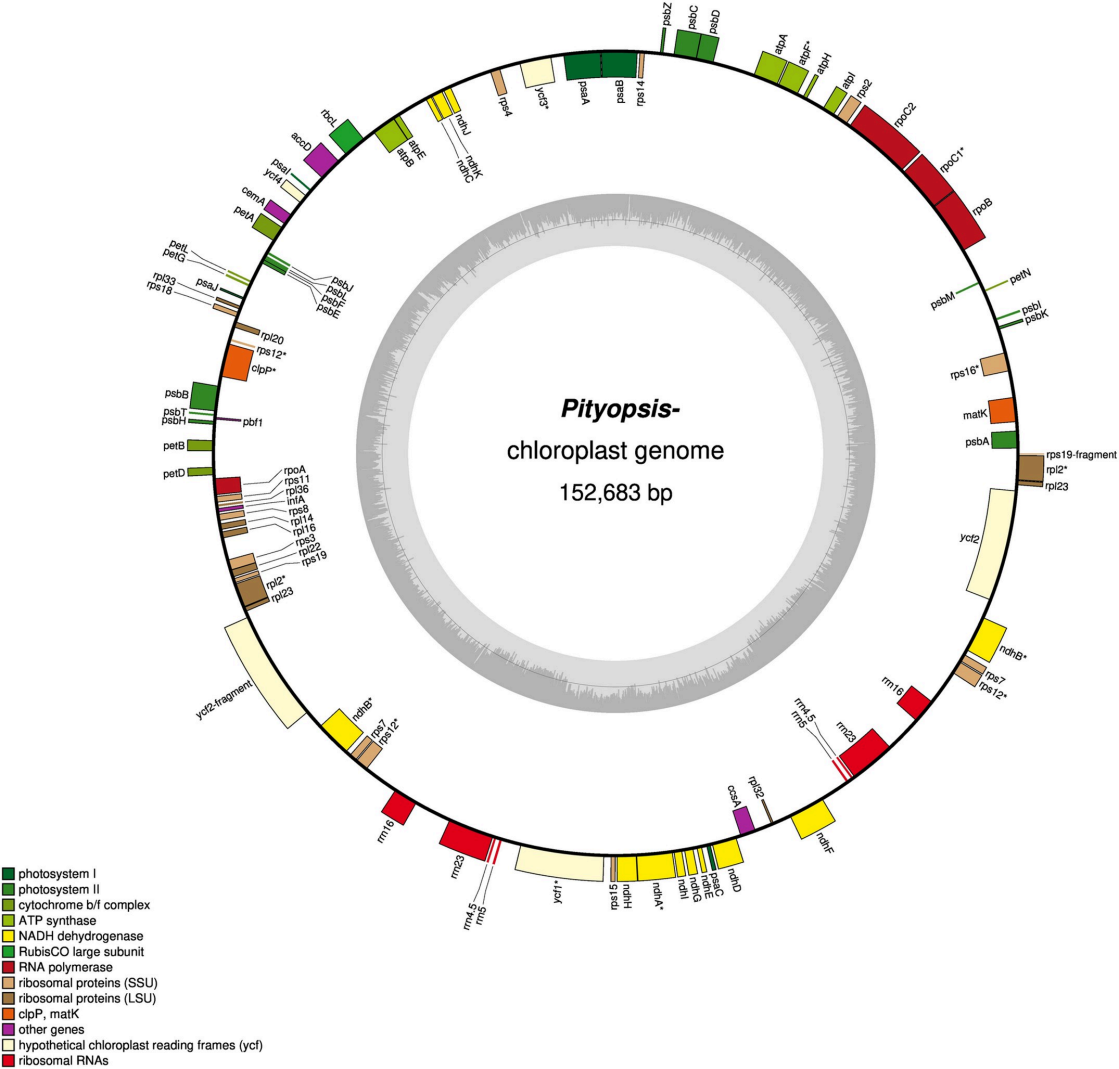
Visualization of *Pityopsis falcata* chloroplast gene map with annotations. The inner circle is GC content. Genes are color coded based on function as per the legend. Genes on the inside of the outer circle are minus (-) strand and genes on the outside of the outer circle are plus (+) strand.

The reference *Pityopsis* cp genome from *P*. *falcata* is a single, circular chromosome, with a large single copy (LSC), small single copy (SSC), and two inverted repeat regions (IR) (Fig 1). The *P*. *falcata* reference was 152,683 bp in length; the LSC was 84,377 bp in length, the SSC was 20,144 bp in length, and the two IRs were 24,081 bp in length. 113 unique genes were identified: 29 transfer RNA (tRNA) genes, 4 ribosomal RNA (rRNA) genes, and 80 protein-coding genes. The IR regions each contained four rRNAs, seven tRNAs, and seven protein-coding genes. Genes directly related to photosynthesis accounted for 42% of all gene function. All assembled *Pityopsis* cp genomes shared synteny with one another and included the same gene features. No inversions or genome rearrangements were apparent in the *Pityopsis* cp genome when compared to each other or other Asteraceae species. The *Pityopsis* cp genome length (152,683 bp) was comparable to cp genomes of other Asteraceae species such as *Lactuca sativa* (lettuce) and *Jacobaea vulgaris*, although the LSC and SSC were longer than those of other species (Table 2). Asteraceae cp genomes contain approximately 114 genes according to Wang *et al*. [36]; we identified 113 genes from the *Pityopsis* cp genome. When including genes duplicated in the IRs, 131 genes were identified, of which 87 were protein-coding. This is five genes fewer than found (including duplicates) in *J*. *vulgaris* [37]. Functional groups of genes were all appropriately represented in the *Pityopsis* cp genomes as compared to those of *Aster spathulifolius* [38], with all anticipated protein-coding genes seen in *Pityopsis*. All photosynthesis system I and II genes expected in angiosperms were seen, as compared to the list from Wakasugi *et al*. [39].

**Table 2:** Comparison of the *Pityopsis falcata* chloroplast genome to other Asteraceae species.

	*Pityopsis falcata*	*Aster spathulifolius*	*Chrysanthemum indicum*	*Helianthus annuus*	*Jacobea vulgaris*	*Lactuca sativa*
Length (bp)	152,683	149,473	150,972	151,104	150,686	152,772
LSC (bp)	84,377	81,998	82,740	83,530	82,855	84,105
SSC (bp)	20,144	17,973	18,394	18,308	18,258	18,599
IR (bp)	24,081	24,751	24,971	24,633	25,000	25,034
No. genes	113	111	114	115	115	115
No. protein-coding genes	80	78	80	81	81	78
No. tRNAs	29	29	30	30	30	30
No. rRNAs	4	4	4	4	4	4
Duplicated genes	18	18	18	18	18	18

We included a single IR in the Mauve alignment and pairwise analyses. Percent identity was higher between cp genomes than the nuclear sequences. Pairwise percent identity was calculated for all 12 *Pityopsis* cp genomes and the outgroup, *H*. *annuus* (Table 3). The most similar cp genomes based on percent identity were *P*. *graminifolia* var. *latifolia* and *P*. *aspera* var. *aspera* (Table 3). The taxa with the most similar nuclear ribosomal regions were *P*. *aspera* var. *adenolepis* and var. *aspera* (99.83%), *P*. *aspera* var. *adenolepis* and *P*. *graminifolia* var. *graminifolia* (99.83%), and *P*. *aspera* var. *adenolepis* and *P*. *graminifolia* var. *latifolia* (99.84%) (Table 3).

**Table 3:** Pairwise alignment comparison of 12 *Pityopsis* species and varieties. Below diagonal is pairwise percent identity between chloroplast genomes calculated from a Mauve multiple sequence alignment. Above diagonal is pairwise percent identity between the short nuclear ribosomal region of the same species, calculated from a MUSCLE multiple sequence alignment.

	1	2	3	4	5	6	7	8	9	10	11	12	13
*Helianthus annuus*		77.72	77.73	77.66	77.69	77.75	77.69	77.74	77.72	77.69	77.76	77.73	77.62
*P*. *aspera* var. *adenolepis*	87.40		99.83	99.44	98.77	98.66	99.83	99.84	98.65	99.71	99.36	98.67	99.33
*P*. *aspera* var. *aspera*	87.47	99.65		99.33	98.66	98.56	99.72	99.90	98.53	99.72	99.23	98.55	99.21
*P*. *falcata*	87.41	99.56	99.66		98.78	98.71	99.44	99.36	98.71	99.28	99.17	98.73	99.49
*P*. *flexuosa*	87.49	99.61	99.70	99.62		99.11	98.76	98.71	99.12	98.65	98.94	99.15	98.76
*P*. *graminifolia* var. *aequilifolia*	87.53	99.73	99.86	99.75	99.76		98.64	98.57	99.74	98.48	99.12	99.72	98.65
*P*. *graminifolia* var. *graminifolia*	87.40	99.62	99.66	99.62	99.65	99.79		99.75	98.61	99.67	99.34	98.63	99.39
*P*. *graminifolia* var. *latifolia*	87.52	99.67	99.97	99.65	99.72	99.86	99.69		98.57	99.70	99.27	98.59	99.25
*P*. *graminifolia* var. *tenuifolia*	87.44	99.54	99.86	99.55	99.60	99.74	99.77	99.86		98.47	99.11	99.77	98.64
*P*. *graminifolia* var. *tracyi*	87.45	99.65	99.68	99.61	99.63	99.75	99.83	99.66	99.77		99.19	98.49	99.16
*P*. *oligantha*	87.51	99.75	99.77	99.73	99.77	99.86	99.72	99.78	99.66	99.78		99.13	99.06
*P*. *pinifolia*	87.51	99.60	99.72	99.63	99.86	99.77	99.68	99.72	99.65	99.67	99.75		98.62
*P*. *ruthii*	87.50	99.66	99.72	99.66	99.69	99.80	99.65	99.71	99.61	99.71	99.80	99.70	

### Phylogenetic analyses

**Fig 2:**
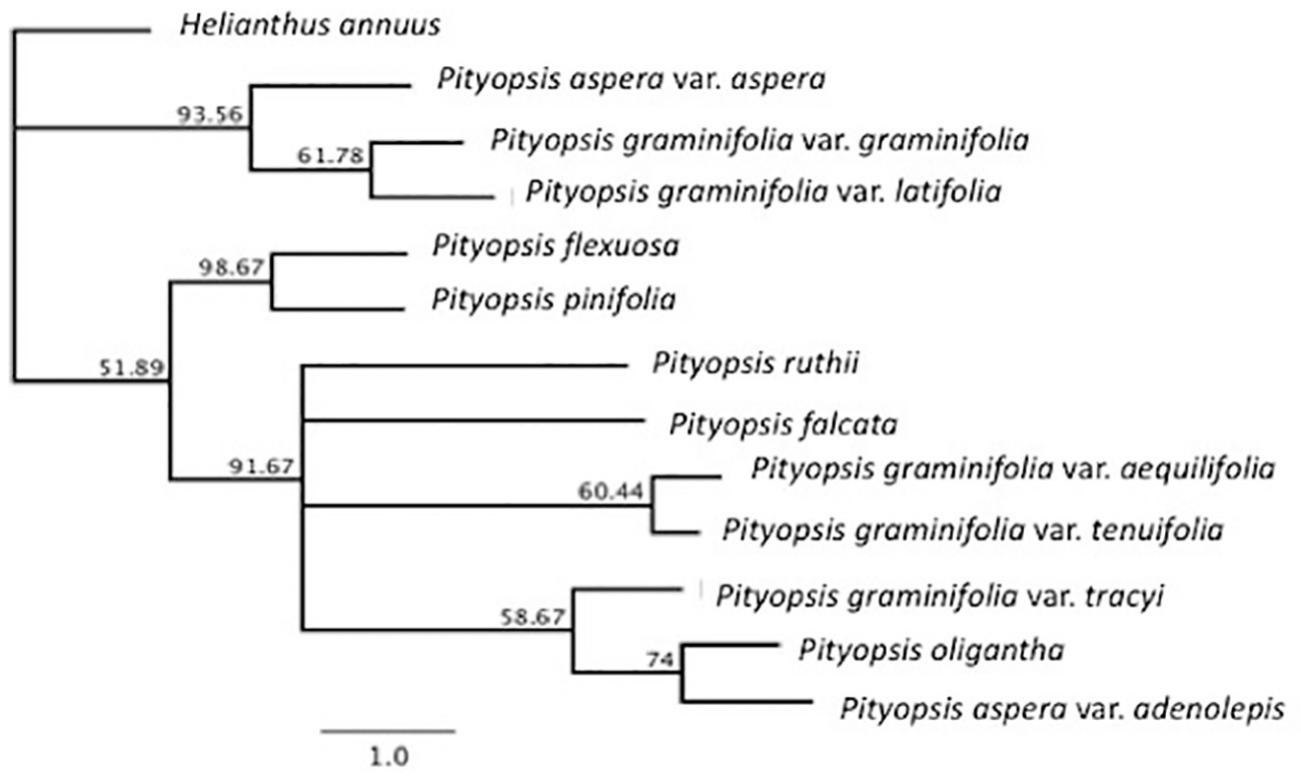
Phylogenetic relationships within *Pityopsis* using the maximum likelihood approach from whole chloroplast genomes, with *Helianthus annuus* as the outgroup. The tree was constructed using the GTR + GAMMA model within RAxML with 1000 replicates. A consensus tree was built using a 50% support threshold. Numbers above nodes are bootstrap values (> 50%).

**Fig 3:**
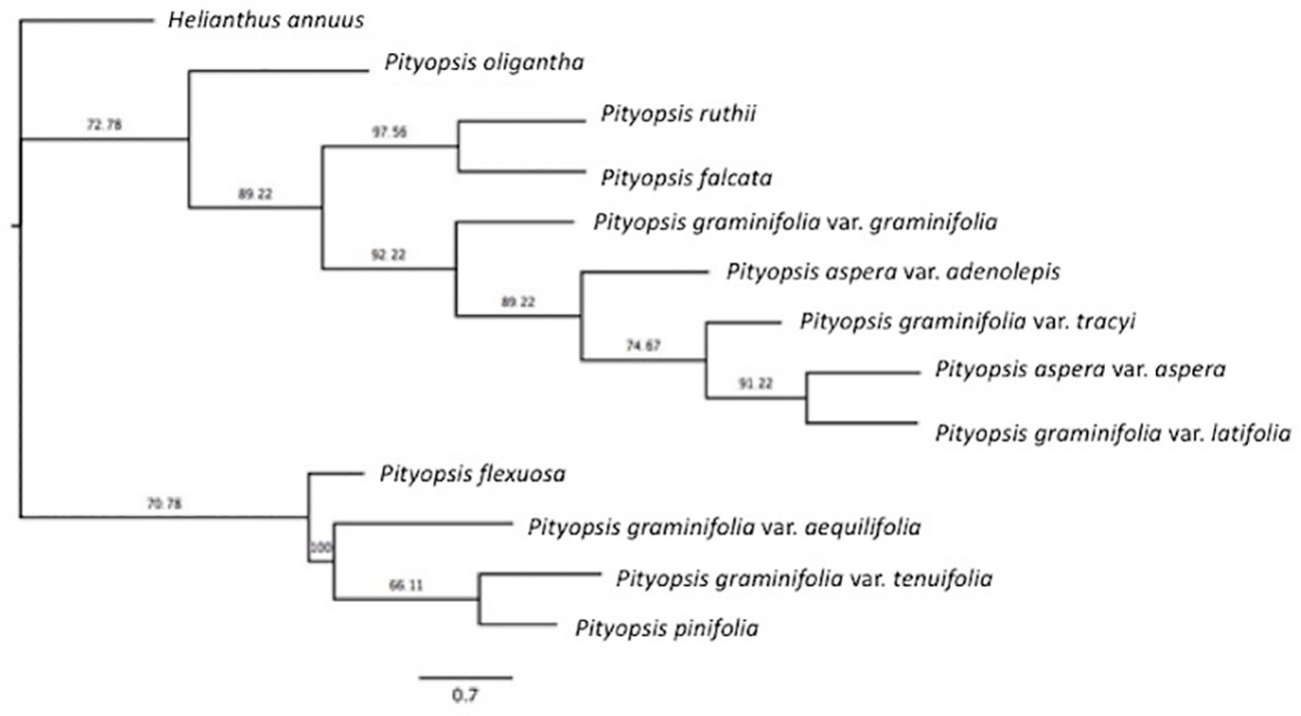
Phylogenetic tree constructed from nuclear ribosomal region of all sampled *Pityopsis* taxa with related species *Helianthus annuus* as an outgroupusing the maximum likelihood approach. The tree was constructed using the GTR + GAMMA model within RAxML with 1000 replicates. A consensus tree was built using a 50% support threshold. Numbers above nodes are bootstrap values (> 50%).

We reconstructed two maximum likelihood (ML) phylogenies, one with cp genomes (Fig 2) and one using the nuclear ribosomal repeat region (Fig 3). Only branches with 50% bootstrap (BS) support or higher were included in the topology. The cp genome and nuclear ribosomal region ML phylogenies differ primarily in the placement of some varieties of *P*. *graminifolia* and the two varieties of *P*. *aspera*. A close relationship between *P*. *pinifolia* and *P*. *flexuosa* was supported in both phylogenies, with the chloroplast tree showing the two as sister species (BS > 98). A similar relationship between *P*. *graminifolia* var. *aequilifolia* and var. *tenuifolia* was moderately supported in the nuclear (BS = 80.6) but weakly supported in the chloroplast tree (BS = 61.8). *P*. *falcata* and *P*. *ruthii* were placed near one another (BS = 91.67) in the chloroplast tree, and the two were placed as sister species in the nuclear phylogenetic tree with strong support (BS = 97.6). The placement of the two *P*. *aspera* varieties is incongruent, with the chloroplast tree showing divergence and the nuclear tree supporting (BS = 89.2) a closer relationship. The placement of *P*. *oligantha* was also incongruent between the two trees.

## Discussion

In this study, we examined relationships among *Pityopsis* species using whole genome sequencing to assemble and compare whole chloroplast genomes. All twelve complete *Pityopsis* cp genomes displayed attributes common among angiosperm cp genomes, with quadripartite structure including the LSC, SSC, and a pair of inverted repeats (IRa and IRb). Although there were no genomic rearrangements apparent and gene order was maintained, the sizes of the cp genomes ranged from 152,558 to 152,747, suggesting small genetic differences.

The *Pityopsis* cp genome included 29 unique tRNA genes, comparable to *A*. *spathulifolius* (29) and *J*. *vulgaris* (29). Within Asteraceae, 29 tRNA genes per cp genome is typical [36,40]. The number of rRNA genes found in the IR of *Pityopsis* is consistent with the number found in several other Asteraceae species, including *A*. *spathulifolius* [38], *H*. *annuus* and *L*. *sativa* [40], and *J*. *vulgaris* [37]. The *ycf1* and *ndhH* genes in *Pityopsis* did not overlap, consistent with *H*. *annuus* and other species within Heliantheae, rather than overlapping as seen in Astereae species such as *A*. *spathulifolius* [38]. Additionally, the *ycf15* gene was present in *Pityopsis* cp genomes, a phenomenon that distinguishes *H*. *annuus* from *Chrysanthemum indicum*, *C*. × *morifolium*, and *Guizotia abyssinica*, in which *ycf15* is absent [36]. We do not know whether *ycf15* is co-transcribed with trnL-CAA and *ycf2*, as in *Camellia* [41], but all three genes are present in *Pityopsis* cp genomes. Due to the phylogenetic relationship between *Pityopsis* and *H*. *annuus*, we expected to see similarities with *Helianthus*, such as presence of *ycf15*, rather than similarities with the more distantly related genera such as *Chrysanthemum*.

The close relationship between *P*. *flexuosa* and the varieties of *P*. *graminifolia* seen in a previous study [4] was not evident in the whole cp genome phylogeny, or the nuclear ribosomal region phylogeny. Our findings are also not consistent with the division of the genus into the sections of *Graminifoliae* and *Pityopsis* proposed by Semple and Bowers [13]. In the nuclear and chloroplast phylogenetic trees section, *Pityopsis* is separated by species within *Graminifoliae*. Both sets of trees placed *P*. *ruthii* and *P*. *falcata* close together as sister species and did the same with *P*. *flexuosa* and *P*. *pinifolia*. The two species with the most disagreement between datasets are both tetraploids, *P*. *aspera* var. *adenolepis* and *P*. *oligantha*. This incongruence might be explained by the difference in inheritance for the two datasets, with the chloroplast inherited maternally and the ribosomal region biparentally; in allopolyploids, this means inheriting the ribosomal region from two different species. Resolution of the contributions to the incongruent placement of polyploid species within *Pityopsis* will require expanded sampling at the population level beyond the scope of the current study.

It is not confirmed whether *Pityopsis* polyploids are auto- or allopolyploids, though there is some evidence that allopolyploidy is the mechanism of genome duplication in *P*. *graminifolia* var. *latifolia*, and that polyploid varieties within *Pityopsis* may be allopolyploid hybrids of other species and varieties [4]. The incongruence between the nuclear and cp trees in the placement of *P*. *aspera* varieties suggest there has been cp transfer through hybridization involved, likely involving *P*. *aspera* var. *adenolepis* and *P*. *oligantha*. Our results do not provide evidence to refute Nesom’s [2] categorization of the two varieties as separate species based on morphology and distribution: *P*. *aspera* and *P*. *adenolepis*. The differences between our nuclear and chloroplast phylogenies also support the hypothesis that *P*. *oligantha* is allopolyploid.

Incongruences between nuclear and chloroplast datasets support allopolyploidy in both *P*. *oligantha* and *P*. *aspera* var. *adenolepis*. *P*. *graminifolia* var. *tracyi* is also a possible allopolyploid, as the hexaploid is placed differently in the two phylogenies, with it placed in the same clade as *P*. *graminifolia* var. *latifolia* and both *P*. *aspera* varieties in the nuclear phylogenies but with *P*. *oligantha* and only *P*. *aspera* var. *adenolepis* in the chloroplast phylogeny. Our results support further investigation into the *P*. *graminifolia* complex, as the varieties were not placed together in either phylogeny, supporting a reorganization of the species, particularly for *P*. *graminifolia* var. *aequilifolia* and var. *tenuifolia* which were placed closer to one another than to other *P*. *graminifolia* varieties in both trees. Indeed, based on morphology and distribution, Nesom [2] names the varieties as distinct species from *P*. *graminifolia*: *P*. *aequilifolia* and the more widespread *P*. *tenuifolia*. The cp genomes from all species and varieties of *Pityopsis* will provide information to future researchers interested in the genus or speciation in plants. Although our study presents the most complete molecular dataset for *Pityopsis* to date, sampling inconsistencies between morphological and molecular studies may contribute to taxonomic confusion.

The variation among chloroplast genomes of *Pityopsis* species provide a mechanism of distinguishing between species and varieties for use in future studies, as well as a broader of understanding diversity within the genus. We have assembled whole chloroplast genomes that will allow further study of individual species as well, opening possibilities for future work in chloroplast transcriptomics, furthering knowledge of variable regions within the chloroplast, and providing information for future studies of *Pityopsis* and Asteraceae.

## Supporting information

S1 Table Statistics from original genomic sequences for all *Pityopsis* individuals. (DOCX)

S2 Table Statistics of *Pityopsis* sequences mapped to the *Helianthus annuus* chloroplast reference genome using Bowtie2. (DOCX)

S3 Table Pairwise alignment comparison of 12 *Pityopsis* species and varieties. Below diagonal is pairwise percent identity between chloroplast genomes calculated from a Mauve multiple sequence alignment. Above diagonal is pairwise percent identity between the short nuclear ribosomal region of the same species, calculated from a MUSCLE multiple sequence alignment.
(XLSX)
