# Supplementary material for: Complete chloroplast genome comparisons for Pityopsis (Asteraceae)
Source: PLoS One. 2020 Dec 28;15(12):e0241391. doi: 10.1371/journal.pone.0241391 (PMC7769439; doi:10.1371/journal.pone.0241391)
Supplement: S1 Table — (DOCX) [file pone.0241391.s001.docx]

| Table S1: Statistics from original genomic sequences for all *Pityopsis* individuals | | | | |
| --- | --- | --- | --- | --- |
| Species | Avg. contig length (bp) | No. reads | Total bp | % GC |
| *P. aspera* var. *adenolepis* | 231.80 | 3,780,160 | 876,251,906 | 0.50 |
| *P. aspera* var. *aspera* | 236.90 | 15,363,629 | 3,640,739,774 | 0.35 |
| *P. falcata* | 235.94 | 4,448,332 | 1,049,529,236 | 0.36 |
| *P. flexuosa* | 218.68 | 5,245,587 | 1,149,603,092 | 0.51 |
| *P. graminifolia* var. *aequilifolia* | 170.16 | 33,339,900 | 567,294,195 | 0.35 |
| *P. graminifolia* var. *graminifolia* | 219.22 | 11,416,970 | 2,502,798,353 | 0.36 |
| *P. graminifolia* var. *latifolia* | 231.77 | 3,553,331 | 823,569,125 | 0.35 |
| *P. graminifolia* var. *tenuifolia* | 196.40 | 7,276,227 | 1,429,009,580 | 0.47 |
| *P. graminifolia* var. *tracyi* | 230.27 | 4,950,830 | 1,140,021,069 | 0.36 |
| *P. oligantha* | 232.32 | 3,451,455 | 801,833,869 | 0.38 |
| *P. pinifolia* | 237.57 | 5,863,119 | 1,391,194,961 | 0.35 |
| *P. ruthii* | 73.60 | 5,529,160 | 40,697,5026 | 0.36 |
| All species and varieties were sequenced using the Illumina MiSeq platform. | | | | |
