# Supplementary material for: Complete chloroplast genome comparisons for Pityopsis (Asteraceae)
Source: PLoS One. 2020 Dec 28;15(12):e0241391. doi: 10.1371/journal.pone.0241391 (PMC7769439; doi:10.1371/journal.pone.0241391)
Supplement: S2 Table — (DOCX) [file pone.0241391.s002.docx]

| Table S2. Statistics of *Pityopsis* sequences mapped to the *Helianthus annuus* chloroplast reference genome using Bowtie2 | | | | |
| --- | --- | --- | --- | --- |
| Species | No. mapped reads | No. bp mapped | % mapped | Coverage |
| *P. aspera* var*. adenolepis* | 22,554 | 4,109,187 | 0.49 | 28.34x |
| *P. aspera* var. *aspera* | 123,750 | 34,872,370 | 1.09 | 240.50x |
| *P. falcata* | 134,967 | 28,365,271 | 2.55 | 195.62x |
| *P. flexuosa* | 23,800 | 5,682,784 | 0.40 | 39.19x |
| *P. graminifolia* var. *aequilifolia* | 6,571 | 762,368 | 0.29 | 5.26x |
| *P. graminifolia* var. *graminifolia* | 63,019 | 12,113,523 | 1.28 | 83.54x |
| *P. graminifolia* var*. latifolia* | 55,981 | 12,950,819 | 1.36 | 89.32x |
| *P. graminifolia* var. *tenuifolia* | 18,778 | 17,958,347 | 1.05 | 123.85x |
| *P. graminifolia* var*. tracyi* | 140,727 | 27,133,639 | 2.29 | 187.13x |
| *P. oligantha* | 12,546 | 2,306,733 | 0.30 | 15.91x |
| *P. pinifolia* | 26,986 | 6,582,877 | 0.57 | 45.40x |
| *P. ruthii* | 199,621 | 16,237,978 | 3.44 | 111.99x |
| All species and varieties were sequenced using the Illumina MiSeq platform. | | | | |
